# Association between leukemic immunophenotype and overall survival in patients with acute promyelocytic leukemia: a retrospective cohort study

**DOI:** 10.3389/fcell.2026.1747649

**Published:** 2026-01-14

**Authors:** Xuan Zha, Li Ma, Yun Yue, XiaoYu Wei, Baolan Sun, Weiguo Wang

**Affiliations:** 1 Department of Laboratory Medicine, Affiliated Hospital of Nantong University, Nantong, Jiangsu, China; 2 Department of Laboratory Medicine, Fu Yang People’s Hospital, Fuyang, Anhui, China

**Keywords:** acute promyelocytic leukemia, flow cytometry, immunophenotype, overall survival, PML- RARα fusion gene

## Abstract

**Introduction:**

Acute promyelocytic leukemia (APL) is a distinct subtype of acute myeloid leukemia (AML) with unique clinical features. Flow cytometry (FCM) immunophenotyping analysis is crucial for accurate diagnosis and prognostic stratification. This study aims to explore the association between specific immune phenotype markers in APL patients and overall survival (OS).

**Methods:**

In this retrospective cohort study, immunophenotypic data from 72 APL patients were analyzed by FCM. Continuous and categorical variables are presented as mean ± standard deviation and frequency (percentage), respectively. Group comparisons were performed using ANOVA and Chi-square tests. Cox proportional hazards models were used to identify prognostic factors for OS, with results expressed as hazard ratios (HRs) and 95% confidence intervals (CIs). Kaplan-Meier survival analysis was employed to assess the impact of CD56, CD2, CD34, and CD200 expression on OS. Subgroup analyses were conducted based on age, gender, white blood cell count (WBC), and disseminated intravascular coagulation (DIC).

**Results:**

The baseline age (p = 0.513) and gender (p = 0.881) were comparable across different PML-RARα isoform groups. Compared to non-APL AML, APL blasts were characterized by significantly higher expression of CD33, CD13, CD9, and MPO (all p < 0.05), and lower expression of CD7, CD34, CD56, CD38, CD200, and HLA-DR (all p < 0.001). The PML-RARα (S-type) group showed relatively higher expression of CD34, CD2, and CD200 than the L-type group. Univariate Cox analysis revealed that expression of CD34, CD2, CD56, and CD200 were all significantly associated with poorer OS. After multivariate adjustment, CD2 (adjusted HR = 1.04, 95% CI: 1.01–1.07, p = 0.004) and CD200 (adjusted HR = 1.04, 95% CI: 1.01–1.06, p = 0.009) remained independent adverse prognostic factors. Subgroup analysis confirmed that the negative prognostic impact of CD2 and CD200 expression was consistent across different patient subgroups.

**Conclusion:**

Compared with non-APL-AML patients, APL patients (PML-RARα (S-type) and PML-RARα (L-type)) exhibit unique immunophenotypic changes. The expression frequencies of CD56, CD2, CD34, and CD200 in leukemia cells are significantly correlated with the OS of APL patients, and the high expression of these indicators before treatment may be an adverse prognostic factor for APL patients.

## 1 Introduction

Acute Promyelocytic Leukemia (APL), a distinct subtype of AML characterized by the t (15; 17) (q24; q21) translocation generating the PML-RARA fusion oncogene, represents a medical emergency due to its high risk of fatal coagulopathy (de Thé et al., 1990). Although it constitutes only 5%–15% of AML cases, APL is a critical focus in hematology due to its remarkable curability with targeted therapies like all-trans retinoic acid (ATRA) and arsenic trioxide (ATO), achieving long-term remission rates exceeding 90% (Sanz et al., 2019; Wang et al., 2022; Yun, 2025). Despite these advances, early mortality remains a significant challenge, primarily driven by hemorrhage and differentiation syndrome, highlighting the critical need for rapid and accurate diagnosis (Avvisati et al., 2001; Dhakal et al., 2021; Infante et al., 2023). Timely identification is paramount for initiating targeted therapy and preventing catastrophic complications.

Immunophenotyping by FCM is indispensable in preliminary diagnostic workup of AML, including APL. APL blasts typically exhibit a characteristic immunophenotype: high side scatter (SSC), strong expression of CD33 and CD13, frequent lack of HLA-DR and CD34, and variable expression of CD117 (Orfao et al., 1999; Ally and Chen, 2024). The PML-RARA fusion exists in three major isoforms (bcr1/L-type, bcr3/S-type, bcr2/V-type), with L-type being the most common. Some studies have shown that subtype types may be associated with clinical characteristics or outcomes (Bradstock et al., 1994). For example, studies have indicated that in APL patients with the bcr3/S-type subtype, the initial response of their leukemia cells to ATRA may differ from that of patients with the bcr1/L-type subtype (Huang et al., 2024). This difference may be related to subtle variations in immune phenotypes among different subtypes, but the specific mechanism still requires in-depth research (Liquori et al., 2020). Beyond the core phenotype, the expression of ‘aberrant’ antigens, such as CD2, CD56, CD34, CD11b, CD15, CD7, and CD25, has been increasingly reported in APL subsets (Xu et al., 2014; Fang et al., 2022). Recent investigations have explored the potential diagnostic utility of these aberrant markers, particularly in differentiating APL from other AML subtypes, and their prognostic significance regarding relapse risk or early mortality (Senapati et al., 2025). DIC is a common critical complication caused by APL. In this study, five APL patients died early within 30 days, among whom four died due to the occurrence of DIC. Some studies have mentioned that CD56 and CD34 are associated with the occurrence of DIC in APL (Li P. et al., 2024). CD56 and CD2 have also been found to be related to poor outcomes in APL (Lin et al., 2004; Ono et al., 2011; Kandeel et al., 2020; Siraj et al., 2021). The prognostic value of comprehensive immunophenotypic profiles, particularly with respect to specific PML-RARA isoforms and survival endpoints, requires further robust validation in larger, well-characterized patient populations.

Despite established diagnostic patterns, significant gaps persist. The precise prognostic impact of specific aberrant immunophenotypes, particularly in the context of modern ATRA/ATO regimens, remains incompletely defined. Previous studies often yielded conflicting results regarding associations between specific markers (e.g., CD2, CD56, CD34) and outcomes like relapse-free survival (RFS) or OS, potentially due to cohort heterogeneity, treatment variations, and limited sample sizes (Lin et al., 2004; Ono et al., 2011; Fang et al., 2022). Furthermore, the relationship between these immunophenotypic variations and the underlying PML-RARA isoform types warrants in-depth investigation to determine whether isoform-specific biological differences are reflected in surface marker expression and clinical behavior (Liquori et al., 2020). Concurrently, there is a need to identify novel immunophenotypic predictors of early mortality risk. By analyzing a comprehensive panel of markers within a well-defined cohort, we aim to provide reliable evidence clarifying the auxiliary diagnostic value and independent prognostic significance of leukemic immunophenotypes in APL, ultimately contributing to improve risk stratification and patient management.

## 2 Materials and methods

### 2.1 Study population

**FIGURE 1:**
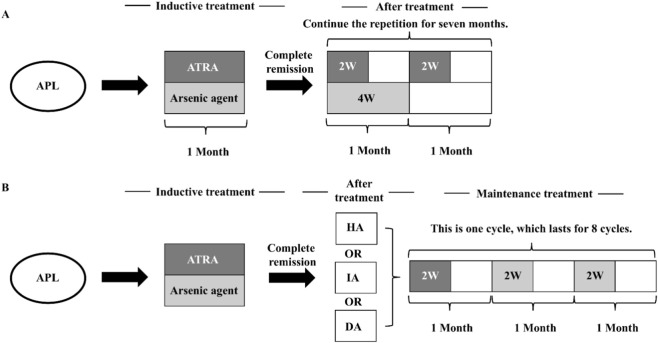
Therapeutic regimen of the APL (Chinese Society of Hematology, Chinese Medical Doctor Association, 2018). **(A)** Treatment process of ATRA + arsenic agent regimen. **(B)** Treatment process of ATRA + arsenic agent + other chemotherapy regimens. (HA regimen: Homoharringtonine 2 mg·m^−2^ d^−1^, days 1–7; Ara-C 100 mg·m^−2^ d^−1^, days 1–5. IA regimen: Idarubicin 8 mg·m^−2^ d^−1^, on days 1–3; Ara-C 100 mg·m^−2^ d^−1^, on days 1–5. DA regimen: Daunorubicin (DNR) 40 mg·m^−2^ d^−1^, on days 1–3; Ara-C 100 mg·m^−2^ d^−1^, on days 1–5.).

We retrospectively collected data from 453 AML patients treated at Fuyang People’s Hospital from January 2014 to March 2023, including 72 APL patients (37 males and 35 females) with an average age of 45.6 ± 14.3 years old. The non-APL-AML group comprised 381 patients (242 males and 139 females) with an average age of 58.9 ± 17.7 years old. The diagnosis of AML was based on the criteria established by the World Health Organization (WHO) classification of tumors of hematopoietic and lymphoid tissues (Arber et al., 2016). Patients with APL received a unified treatment plan in accordance with the Chinese Guidelines for the Diagnosis and Treatment of Acute Promyelocytic Leukemia (2018) (Chinese Society of Hematology, Chinese Medical Doctor Association, 2018). High-risk patients were often treated with combined chemotherapy (Figure 1). This study was approved by the Ethics Committee of Fuyang People’s Hospital, and all patients provided informed consent at the start of therapy.

### 2.2 Specimen preparation

Bone marrow or peripheral blood samples (2–3 mL) were collected in heparin-containing tubes. Fluorescein-labeled monoclonal antibody (clones in parenthesis) were fluorescein isothiocyanate (FITC), phycoerythrin (PE), Peridinin-Chlorophyll-Protein Complex (PerCP) and Allophycocyanin (APC), included: CD7 (4H9), CD117 (104D2), CD33 (P67.6), CD10 (HI10a), CD34 (8G12), CD64 (10.1), CD13 (L138), CD11b (D12), CD2 (S5.2), CD123 (9F5), CD4(SK3), CD25 (2A3), CD15 (HI98), CD56 (NCAM16.2), HLA-DR (L243), CD200 (MRC OX-104), CD9 (M-L13), CD14 (MφP9), MPO (5B8), CD79a (HM47), CD3 (SK7), CD45 (2D1). Fix membrane rupturing agent, lysis solution, and FACS-Calibur flow cytometer were all purchased from BD Biosciences (San Jose, United States).

### 2.3 Flow cytometry

The bone marrow and blood cells were incubated with antibodies for a duration of 30 min at room temperature, followed by the lysis of erythrocytes using a standard lyse/wash technique. The antigenic expression in blast cells was systematically analyzed through multi-parametric flow cytometry (BD FACSCalibur, United States) utilizing Cellquest software. Gating was performed using CD45/SSC, mature lymphocytes and mature granulocytes in the specimen serving as the internal reference. The determination of cell surface antigen expression was considered positive if the analyzed events demonstrated a level of expression ≥ 20%, while a level of expression ≥ 80% was classified as high expression 14. For cytoplasmic antigen expression, a level of expression ≥ 10% was considered positive based on the analyzed events15.

### 2.4 Follow up

The survival of active patients was assessed through telephone interviews, outpatient follow-up appointments, and case reviews. The final follow-up was conducted on March 1, 2023. The 5-year OS was defined as the period between the initial diagnosis and either death or the conclusion of the follow-up period.

### 2.5 Statistical analysis

Statistical analysis was conducted using FreeStatisticsV2.2.0. Continuous variables were presented in the form of mean ± standard deviation, while categorical variables were presented in the form of frequency (percentage). Inter-group comparisons for continuous variables were handled using analysis of variance, and for categorical variables, Chi-square tests were employed. The Cox proportional hazards model and Kaplan-Meier survival analysis determined the predictors of survival, presenting HR along with their 95% CIs. The significance of the models was evaluated through Wald tests and likelihood ratio tests. Subgroup analysis and forest plots were used to examine the interaction effects of age, gender, DIC, and WBC. Statistical significance was defined as a two-sided α < 0.05. Unexplained estimates (e.g., infinite confidence intervals) were excluded from the report.

## 3 Results

### 3.1 Baseline characteristics of study subjects

**FIGURE 2:**
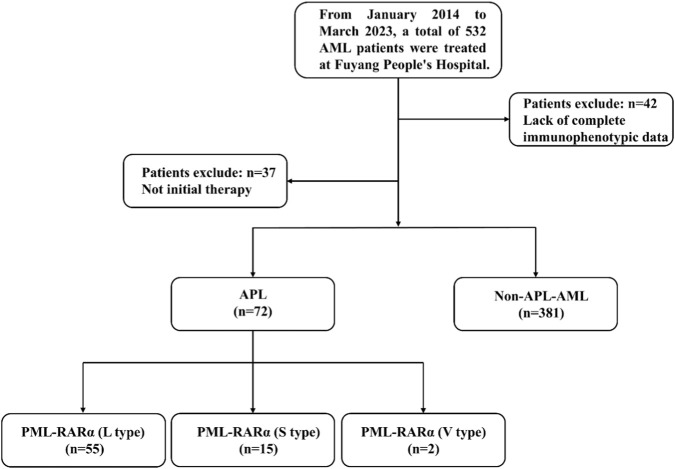
Flowchart of the study.

The cohort construction process of this retrospective analysis is shown in Figure 2.

Among the 532 leukemia patients screened from January 2014 to March 2023, 79 were excluded: 42 patients had incomplete immunophenotypic data, and 37 patients were receiving active treatment. After applying the inclusion criteria, 72 newly diagnosed APL patients and 381 AML patients diagnosed as non-APL (all with complete flow cytometry data) formed the final analysis cohort. As shown in Table 1, the average age of the APL group was 45.6 ± 14.3 years, significantly lower than that of the non-APL-AML group at 58.9 ± 17.7 years (p < 0.001), with an intergroup difference of 13.3 years. The proportion of males in the APL group was 51.4% (37/72), and that of females was 48.6% (35/72). In the non-APL-AML group, the proportion of males was 63.5% (242/381), and that of females was 36.5% (139/381). No significant difference was found between the two groups (p = 0.052). In Table 2, among the 72 APL patients, 55 cases were PML-RARα (L type), 15 cases were PML-RARα (S type), and 2 cases were PML-RARα (V type). There were no significant differences in age (p = 0.513) and gender (p = 0.881) among the groups. While WBC levels differed significantly (p = 0.036), with the median WBC being highest in the S - type subgroup. The incidence of DIC showed no significant difference across groups (p = 0.531). In conclusion, compared with the non-APL AML group, APL patients showed a younger age distribution, but there was no statistical difference in gender distribution between the two groups. And there were no significant differences among the various subtypes of APL in terms of age, gender, and the incidence of complications such as DIC.

**TABLE 1:** Baseline characteristics of participants.

Variables	Total (n = 453)	APL (n = 72)	Non-APL-AML (n = 381)	p
Age, mean ± SD	56.8 ± 17.8	45.6 ± 14.3	58.9 ± 17.7	<0.001
Gender, n (%)				0.052
Males	279 (61.6)	37 (51.4)	242 (63.5)	​
Females	174 (38.4)	35 (48.6)	139 (36.5)	​

**TABLE 2:** Baseline characteristics of APL patients.

Variables	APL (n = 72)	PML-RARα (L-type) (n = 55)	PML-RARα(S-type) (n = 15)	PML-RARα(V-type) (n = 2)	p
Age, mean ± SD	45.6 ± 14.3	44.6 ± 14.0	49.4 ± 15.7	45.0 ± 7.1	0.513
Gender, n (%)		​	​	​	0.881
Males	37 (51.4)	29 (52.7)	7 (46.7)	1 (50)	​
Females	35 (48.6)	26 (47.3)	8 (53.3)	1 (50)	​
WBC, median (IQR)	3.1 (1.2, 7.0)	2.6 (1.1, 5.5)	8.0 (2.6, 19.4)	2.3 (2.2, 2.5)	0.036
DIC, n (%)	​	​	​	​	0.531
Yes	9 (12.5)	6 (10.9)	3 (20)	0 (0)	​
No	63 (87.5)	49 (89.1)	12 (80)	2 (100)	​

### 3.2 Comparison of immunophenotype between APL group and non-APL-AML group

Table 3 shows the expression levels of 20 immune markers in the APL group and the non-APL AML group. The results indicate that compared with the non-APL-AML group, the expression levels of CD33 (p < 0.001), CD13 (p = 0.018), CD9 (p < 0.001), and MPO (p < 0.001) in the APL group were significantly increased, while the expression levels of CD7 (p < 0.001), CD34 (p < 0.001), CD56 (p < 0.001), CD38 (p < 0.001), CD200 (p < 0.001), and HLA-DR (p < 0.001) were significantly decreased. PML-RARα (L-type) was consistent with the immunophenotype of the APL group. Compared with the non-APL AML group, PML-RARα (s-type) mainly showed an increase in the expression of CD33 (p = 0.002), CD2 (p < 0.001), CD9 (p < 0.001), and MPO (p < 0.001), and a decrease in the expression of CD34 (p < 0.001), CD200 (p = 0.034), and HLA-DR (p < 0.001). While compared with PML-RARα (L-type), PML-RARα (s-type) mainly showed an increase in the levels of CD34, CD2, and CD200. The detailed expression of the immunophenotype in each disease group can be found in Supplementary Tables S1–S3.

**TABLE 3:** The expression of 20 types of immune markers in APL group and non-APL-AML group.

Variables	Total (n = 436)	Non-APL-AML (n = 381)	APL (n = 72)	PML-RARα (L-type) (n = 55)	PML-RARα(S-type) (n = 15)	p1	p2	p3	p4
CD7 Median (IQR)	0.0 (0.0, 0.0)	0.0 (0.0, 21.3)	0.0 (0.0, 0.0)	0.0 (0.0, 0.0)	0.0 (0.0, 0.0)	<0.001	<0.001	0.074	0.055
CD117 Mean ± SD	71.0 ± 29.4	72.0 ± 30.3	66.2 ± 20.6	64.2 ± 21.4	69.9 ± 15.1	0.121	0.064	0.785	0.337
CD33 Mean ± SD	68.9 ± 33.2	64.9 ± 33.6	96.0 ± 8.5	96.8 ± 4.5	92.7 ± 16.4	<0.001	<0.001	0.002	0.099
CD10 Mean ± SD	0.1 ± 1.1	0.1 ± 1.2	0.0 ± 0.0	0.0 ± 0.0	0.0 ± 0.0	0.664	0.704	0.843	1
CD34 Median (IQR)	75.8 (0.0, 94.7)	85.1 (27.5, 95.8)	0.0 (0.0, 0.0)	0.0 (0.0, 0.0)	24.8 ± 20.4	<0.001	<0.001	<0.001	<0.001
CD19 Median (IQR)	0.0 (0.0, 0.0)	0.0 (0.0, 0.0)	0.0 (0.0, 0.0)	0.0 (0.0, 0.0)	0.0 (0.0, 0.0)	0.005	0.013	0.193	1
CD13 Mean ± SD	78.6 ± 25.7	77.6 ± 26.7	85.3 ± 16.3	85.5 ± 15.5	83.0 ± 19.6	0.018	0.033	0.437	0.606
CD11b Median (IQR)	0.0 (0.0, 0.0)	0.0 (0.0, 0.0)	0.0 (0.0, 0.0)	0.0 (0.0, 0.0)	0.0 (0.0, 0.0)	<0.001	<0.001	0.177	0.055
CD2 Median (IQR)	0.0 (0.0, 0.0)	0.0 (0.0, 0.0)	0.0 (0.0, 0.0)	0.0 (0.0, 0.0)	24.6 (0.0, 35.6)	<0.001	0.743	<0.001	<0.001
CD123 Mean ± SD	67.8 ± 31.6	68.3 ± 31.7	68.1 ± 30.1	64.5 ± 31.1	80.9 ± 22.6	0.966	0.409	0.127	0.06
CD56 Median (IQR)	0.0 (0.0, 39.4)	0.0 (0.0, 42.9)	0.0 (0.0, 0.0)	0.0 (0.0, 0.0)	0.0 (0.0, 0.0)	<0.001	<0.001	0.124	0.518
CD15 Median (IQR)	0.0 (0.0, 33.7)	0.0 (0.0, 35.1)	0.0 (0.0, 22.7)	0.0 (0.0, 27.8)	0.0 (0.0, 0.0)	0.082	0.366	0.064	0.146
CD14 Median (IQR)	0.0 (0.0, 0.0)	0.0 (0.0, 0.0)	0.0 (0.0, 0.0)	0.0 (0.0, 0.0)	0.0 (0.0, 0.0)	0.018	0.038	0.277	1
CD9 Median (IQR)	24.4 (0.0, 70.7)	0.0 (0.0, 48.0)	97.6 (95.8, 99.0)	97.5 (95.7, 98.9)	97.5 (95.6, 99.2)	<0.001	<0.001	<0.001	0.611
CD38 Mean ± SD	79.6 ± 27.8	81.0 ± 27.9	69.1 ± 25.4	70.2 ± 25.8	62.6 ± 24.5	<0.001	0.007	0.012	0.311
HLA.DR Mean ± SD	65.0 ± 39.4	74.0 ± 33.4	2.3 ± 10.0	2.4 ± 10.6	2.2 ± 8.7	<0.001	<0.001	<0.001	0.959
CD200 Median (IQR)	33.3 (0.0, 77.4)	47.4 (0.0, 84.1)	0.0 (0.0, 0.0)	0.0 (0.0, 0.0)	21.7 (0.0, 39.8)	<0.001	<0.001	0.034	<0.001
CD4 Median (IQR)	0.0 (0.0, 0.0)	0.0 (0.0, 0.0)	0.0 (0.0, 0.0)	0.0 (0.0, 0.0)	0.0 (0.0, 0.0)	<0.001	<0.001	0.072	1
CD25 Median (IQR)	0.0 (0.0, 0.0)	0.0 (0.0, 0.0)	0.0 (0.0, 0.0)	0.0 (0.0, 0.0)	0.0 (0.0, 0.0)	<0.001	<0.001	0.069	1
MPO Median (IQR)	76.1 (18.0, 98.0)	58.5 (12.4, 94.8)	98.7 ± 3.1	99.5 (99.1, 99.8)	99.4 (99.0, 99.6)	<0.001	<0.001	<0.001	0.044

p1: the non-APL-AML, group vs. the APL, group.

p2: the non-APL-AML, group vs. the PML-RARα (L-type) group.

p3: the non-APL-AML, group vs. the PML-RARα (S-type) group.

p4: the PML-RARα (L-type) group vs. the PML-RARα (S-type) group.

### 3.3 Cox multivariate regression analysis analyzed the correlation between immune phenotypes and survival time in APL patients

To explore the association between immune phenotypes and survival rates in APL patients, multivariable Cox regression models were employed (Table 4). In the crude model, CD34 (crude HR = 1.02, 95% CI: 1–1.05, p = 0.042), CD2 (crude HR = 1.05, 95% CI: 1.03–1.08, p < 0.001), CD56 (crude HR = 1.02, 95% CI: 1–1.03, p = 0.012), and CD200 (crude HR = 1.03, 95% CI: 1–1.05, p = 0.025) were significantly associated with survival. After adjusting for age and gender (model Ⅰ), CD2 (adj. HR = 1.05, 95% CI: 1.03–1.08, p < 0.001), CD56 (adj. HR = 1.03, 95% CI: 1.01–1.04, p = 0.001), and CD200 (adj. HR = 1.02, 95% CI: 1–1.05, p = 0.036) remained statistically significant, while the association of CD34 (adj. HR = 1.02, 95% CI: 0.99–1.04, p = 0.144) with survival in APL patients disappeared. Further adjustment for age, gender, and DIC (model Ⅱ) revealed that CD2 (adj. HR = 1.04, 95% CI: 1.01–1.07, p = 0.004) and CD200 (adj. HR = 1.04, 95% CI: 1.01–1.06, p = 0.009) were independently associated with survival rates, whereas other immune phenotypes showed no significant correlations.

**TABLE 4:** Multivariable cox regression models evaluating the relationship between the immune phenotype and the survival rate of APL patients (Due to the extremely low expression levels of CD7, CD10, CD19, CD11b, CD14, HLA-DR, CD4, and CD25 in APL, they were excluded).

Variable	Crude		Model Ⅰ		Model Ⅱ	
	crude.HR (95%CI)	Crude p value	adj.HR (95%CI)	adj.p value	adj.HR (95%CI)	adj.p value
CD117	1 (0.98–1.02)	0.887	1 (0.97–1.02)	0.769	1.02 (0.99–1.04)	0.246
CD33	1.08 (0.93–1.26)	0.333	1.09 (0.93–1.27)	0.297	1.13 (0.92–1.38)	0.242
CD34	1.02 (1–1.05)	0.042	1.02 (0.99–1.04)	0.144	1.01 (0.99–1.04)	0.319
CD13	0.99 (0.96–1.02)	0.43	0.99 (0.96–1.01)	0.288	1.02 (0.98–1.06)	0.284
CD2	1.05 (1.03–1.08)	<0.001	1.05 (1.03–1.08)	<0.001	1.04 (1.01–1.07)	0.004
CD123	1.02 (1–1.04)	0.095	1.01 (0.99–1.03)	0.214	1.01 (0.99–1.04)	0.33
CD56	1.02 (1–1.03)	0.012	1.03 (1.01–1.04)	0.001	1.02 (1–1.03)	0.061
CD15	1.01 (0.99–1.03)	0.224	1.01 (0.99–1.03)	0.151	0.97 (0.95–1)	0.053
CD9	0.95 (0.89–1.03)	0.206	0.95 (0.88–1.04)	0.266	0.95 (0.86–1.05)	0.333
CD38	0.99 (0.97–1)	0.062	0.99 (0.97–1)	0.117	0.99 (0.97–1.01)	0.381
CD200	1.03 (1–1.05)	0.025	1.02 (1–1.05)	0.036	1.04 (1.01–1.06)	0.009
MPO	1.01 (0.84–1.23)	0.893	1.04 (0.84–1.28)	0.722	0.97 (0.83–1.14)	0.714

Crude model: adjusted for none.

Model Ⅰ: adjusted for age, sex.

Model Ⅱ: adjusted for age, sex, and DIC.

### 3.4 Kaplan-Meier survival curves for the impact of CD56, CD2, CD200, and CD34 on the survival time of APL patients

**FIGURE 3:**
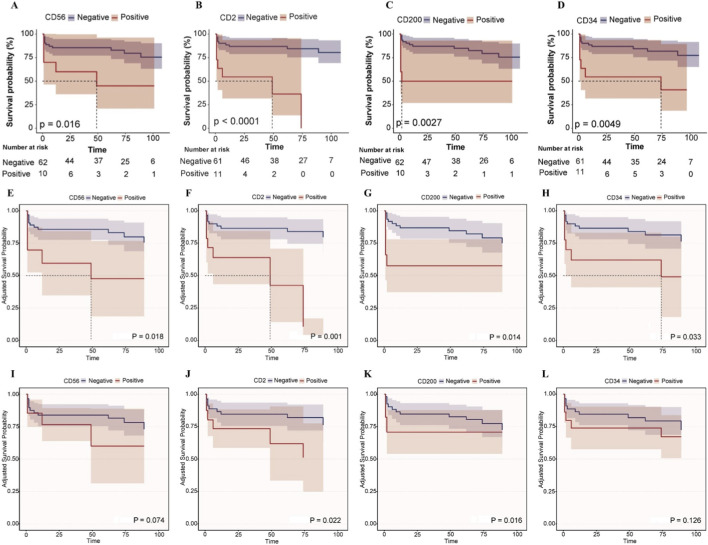
Kaplan-Meier survival curves for the impact of CD56, CD2, CD200, and CD34 on the survival time of APL patients. **(A–D)** Kaplan-Meier survival curves for the impact of CD56, CD2, CD200, and CD34 on the survival time of APL patients. **(E–H)** After adjusting for age and gender, the adjusted Kaplan-Meier survival curves were plotted for the effects of CD56, CD2, CD200 and CD34 on the survival time of APL patients. **(I–L)** After adjusting for age, gender and DIC, the adjusted Kaplan-Meier survival curves were plotted for the effects of CD56, CD2, CD200 and CD34 on the survival time of APL patients.

Figure 3 depicts Kaplan - Meier survival curves illustrating the impact of CD56, CD2, CD200, and CD34 on the survival time of APL patients under different adjustment scenarios. In the unadjusted curves (Figures 3A–D), positive expression of CD56 (p = 0.016), CD2 (p < 0.0001), CD200 (p = 0.0027), and CD34 (p = 0.0049) was significantly associated with worse survival in APL patients. After adjusting for age and gender (Figures 3E–H), positive CD56 (p = 0.018) and CD2 (p = 0.001) remained significantly associated with reduced survival, while CD200 (p = 0.014) and CD34 (p = 0.033) showed a trend of decreased association with survival rate. When further adjusting for age, gender, and DIC (Figures 3I–L), positive CD2 (p = 0.022) and CD200 (p = 0.016) remained significantly associated with reduced survival, and the trends for CD56 (p = 0.074) and CD34 (p = 0.126) towards worse survival persisted albeit non - significantly.

### 3.5 The forest plot shows the subgroup analysis of the effects of CD56, CD2, CD34, and CD200 on the survival time of APL patients

**FIGURE 4:**
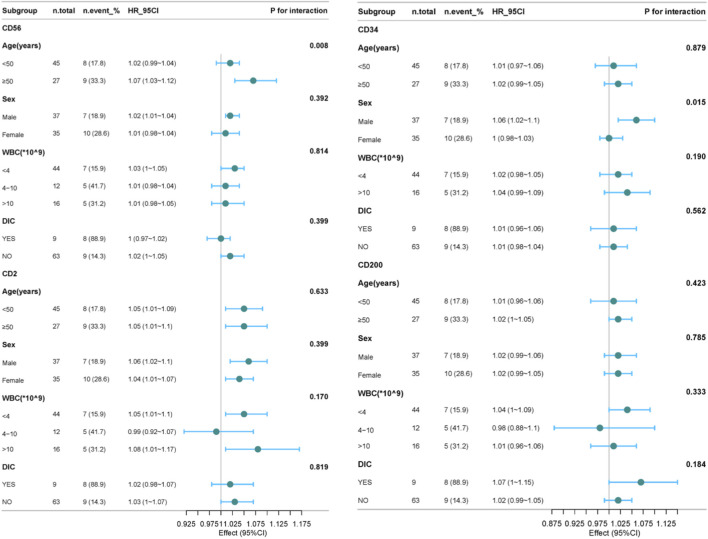
The forest plot shows the subgroup analysis of the effects of CD56, CD2, CD34, and CD200 on the survival time of APL patients. For CD34 has invalid values within the WBC range (4–10) *10^9, it was excluded.

Next, subgroup analyses were conducted to explore the association between leukemia immunophenotypes (CD56, CD2, CD34, CD200) and survival in APL patients, stratified by age, sex, WBC, and DIC status (Supplementary Tables S4–S7). As shown in Figure 4, for CD56, a significant interaction with age was observed (p = 0.008), where the HR was 1.02 (95% confidence interval: 0.99–1.04) in patients younger than 50 years old, and 1.07 (95% confidence interval: 1.03–1.12) in patients older than or equal to 50 years old. It shows that in the subgroup of patients aged ≥50 years, after adjusting for relevant factors, the risk of a significant reduction in survival time for patients with CD56 is significantly higher. For CD34, a significant interaction with gender was observed (p = 0.015), with the hazard ratios being 1.06 (95% confidence interval: 1.02–1.1) for males and 1.0 (95% confidence interval: 0.98–1.03) for females. It is suggested that the impact of CD34 on survival time differs between male and female subgroups, indicating that in the male subgroup, the risk of shortened survival time in patients with CD34 is significantly higher. Besides these, no significant interactions were observed for CD56, CD2, CD34, and CD200 in each subgroup. These results indicate that the relationships between CD56, CD2, CD34, and CD200 and the survival time of APL patients are relatively stable across different subgroups.

## 4 Discussion

APL is a subtype of AML with unique cytomorphological, immunophenotypic, and molecular genetic characteristics. Its pathogenesis is mainly closely related to the formation of the PML-RARα fusion gene caused by chromosomal translocation. In terms of diagnosis, although traditional cytomorphological examination can initially identify the typical promyelocyte morphology of APL, the introduction of immunophenotypic detection has greatly improved the accuracy and specificity of diagnosis. This study systematically analyzed 72 APL patients and 381 non-APL-AML patients, confirming APL’s unique immunophenotypic profile: significantly higher expression of CD33, CD13, CD9, and MPO, and lower expression of CD7, CD34, CD56, CD38, CD200, and HLA-DR compared to non-APL-AML. This aligns with APL’s biological characteristics driven by the PML-RARα fusion gene, which disrupts myeloid differentiation, explaining the distinct marker expression. In addition, this article provides a detailed description of the immunophenotypes in PML-RARα (L-type) and PML-RARα (S-type), and it was found that compared with PML-RARα (L-type), the levels of CD34, CD2 and CD200 in PML-RARα (S-type) were elevated. In terms of prognostic assessment, immunophenotype also plays an important role. Different immunophenotypic characteristics may be closely related to patients' sensitivity to treatment, remission rate, recurrence rate, and long-term survival rate. Based on a retrospective cohort study design, this study aims to deeply explore the association between immunophenotypic characteristics of APL patients and their diagnostic accuracy and prognostic indicators, in order to provide stronger support for clinical accurate diagnosis and the formulation of personalized treatment plans. The results of COX multivariate regression analysis and KM curves showed that CD34, CD2, CD56, and CD200 were significantly correlated with the OS of APL patients. After adjusting for covariates, CD2 and CD200 remained significantly correlated with the OS of APL. Moreover, the results of subgroup analysis showed that the relationships between CD2, CD200 and the survival time of APL patients were relatively stable among different subgroups.

The adverse prognosis associated with specific immunophenotypic markers may be explained by underlying mechanisms related to leukemogenesis and immune evasion. The PML-RARα fusion protein disrupts nuclear bodies and represses gene transcription involved in differentiation and apoptosis, potentially leading to the aberrant expression of markers like CD34 and CD2 (Minami et al., 2025). CD34, a marker of primitive hematopoietic progenitors, is often associated with high-risk disease and increased relapse, possibly reflecting a stem cell-like phenotype with enhanced self-renewal capacity and chemoresistance (Gaafar et al., 2025). CD2, a T-cell marker aberrantly expressed in APL, may promote leukemic cell survival through interactions with the microenvironment or activation of signaling pathways that impede differentiation. Additionally, CD56, a neural cell adhesion molecule, has been linked to increased early mortality, possibly due to its association with coagulation abnormalities like DIC (Murray et al., 1999). In our study, after adjusting for DIC, the association between CD56 and OS disappeared, suggesting that CD56 may contribute to DIC pathogenesis, potentially through enhanced tissue factor expression or endothelial activation. CD200, an immunoregulatory molecule, may suppress T-cell responses and promote immune escape, creating a permissive microenvironment for leukemic cell survival (Baek and Cui, 2024). Multiple researchers have demonstrated that AML patients with high expression of CD200 have significantly shorter survival periods, and anti-CD200 can be used for their treatment (Rastogi et al., 2020; Herbrich et al., 2021). Kandeel EZ et al. also proved that the overexpression of CD200 is an important factor affecting the clinical course of pediatric acute myeloid leukemia (Kandeel et al., 2020). The higher expression of CD34, CD2, and CD200 in the S-type PML-RARα isoform may partly explain the poorer survival observed in these patients, as these markers collectively contribute to a more aggressive disease phenotype (Gupta et al., 2004).

The tumor immune microenvironment plays a critical role in APL progression and treatment response. Our findings of interactions between CD56 and age, and CD34 and gender, suggest that the prognostic impact of these markers may be modulated by host factors and immune context. For example, in males over 50 years, high CD56 and CD34 expression were significantly associated with reduced survival, possibly due to age-related immune senescence or hormonal influences on the microenvironment. Recent studies in AML have highlighted the importance of immune subtypes, such as immune-infiltrated and immune-depleted phenotypes, which correlate with T-cell exhaustion, checkpoint expression, and treatment resistance (Chen et al., 2021; Nixon et al., 2022). In APL, aberrant expression of markers like CD200 may contribute to an immune-suppressive microenvironment by engaging inhibitory receptors on T cells, similar to the PD-1/PD-L1 axis. Targeting these pathways, such as with anti-SLAMF6 antibodies in AML, could represent a promising therapeutic strategy in high-risk APL subsets (Sandén et al., 2025).

This study also has some limitations. Firstly, it established a significant association between the expressions of CD2, CD200, CD34, and CD56 and OS of APL patients, without clarifying the underlying causal mechanisms. The observational design cannot rule out the confounding effects of unmeasured variables (such as genetic background, subtle differences in treatment response) or reverse causality (e.g., whether disease progression itself regulates marker expression). Currently, there is a growing research interest in Mendelian randomization (MR). As a rigorous causal inference framework, MR can address this limitation, and many researchers have drawn highly valuable conclusions through this research paradigm (Li K. et al., 2024; Zhang et al., 2024a; Zhang et al., 2024b; Belbasis et al., 2025; Li et al., 2025). Our future research could also consider adopting a two-sample MR design. MR uses genetic variations (single nucleotide polymorphisms, SNPs) as instrumental variables, which are strongly associated with the expressions of CD2, CD200, CD34, and CD56 (derived from expression quantitative trait locus (eQTL) databases such as eQTLGen). The MR framework will clarify whether these immunophenotypic markers are causal drivers of APL progression or merely associated biomarkers, thereby providing a rigorous basis for the development of targeted therapies and the optimization of risk stratification strategies. Secondly, the subjects of this study all came from a single medical center, and the cohort data were collected retrospectively, which may have selection bias (such as center-specific diagnosis and treatment processes, distribution of patient baseline characteristics, etc.). Therefore, the research results may be more applicable to populations with similar characteristics to the patients in this center. Thirdly, the sample size in this study is relatively small (especially for rare subtypes such as PML-RARα(V-type) APL) and a lack of recurrence/minimal residual disease data. Since PML-RARα(V-type) APL is a rare subtype (accounting for only 8% of PML-RARα positive APL (Slack et al., 2000)), only two cases of PML-RARα(V-type) APL were collected in this study. Due to the difficulty in statistical analysis, we did not include the subgroup of PML-RARα(V-type) APL in the subsequent result analysis.

In the future, we will conduct multi-center, prospective cohort studies, enrolling APL patients from medical institutions of different regions and levels to reduce selection bias and enhance the promotional value and clinical applicability of the research results. Additionally, we will continue to accumulate sample sizes to obtain more reliable clinical data and prognostic evaluations, aiming to verify the conclusions in this study regarding the correlation between immunophenotypic markers and prognosis, and to explore the mechanisms by which these markers affect signaling pathways, immune escape, and therapeutic resistance. Such studies may lead to the discovery of new therapeutic targets, such as the development of prognostic models integrating immunophenotypes.

## 5 Conclusion

Compared with non-APL-AML patients, APL patients (PML-RARα (S-type) and PML-RARα (L-type)) exhibit unique immunophenotypic changes. The expression frequencies of CD56, CD2, CD34, and CD200 in leukemia cells are significantly correlated with the OS of APL patients, and the high expression of these indicators before treatment may be an adverse prognostic factor for APL patients.

## Data Availability

The raw data supporting the conclusions of this article will be made available by the authors, without undue reservation.

## References

[B1] Ally F. Chen X. (2024). Acute myeloid leukemia: diagnosis and evaluation by flow cytometry. Cancers 16 (22), 3855. 10.3390/cancers16223855 39594810 PMC11592599

[B2] Arber D. A. Orazi A. Hasserjian R. Thiele J. Borowitz M. J. Le Beau M. M. (2016). The 2016 revision to the world health organization classification of myeloid neoplasms and acute leukemia. Blood 127 (20), 462–463. 10.1182/blood-2016-06-721662 27069254

[B3] Avvisati G. Lo Coco F. Mandelli F. (2001). Acute promyelocytic leukemia: clinical and morphologic features and prognostic factors. Seminars Hematol. 38 (1), 4–12. 10.1053/shem.2001.20861 11172535

[B4] Baek S. Cui K. (2024). Targeting CD200 in breast cancer: opportunities and challenges in immunotherapeutic strategies. Int. J. Mol. Sci. 26 (1), 115. 10.3390/ijms26010115 39795972 PMC11719565

[B5] Belbasis L. Morris S. van Duijn C. Bennett D. Walters R. (2025). Mendelian randomization identifies proteins involved in neurodegenerative diseases. Brain 148 (7), 2412–2428. 10.1093/brain/awaf018 40037332 PMC12233555

[B6] Bradstock K. Matthews J. Benson E. Page F. Bishop J. (1994). Prognostic value of immunophenotyping in acute myeloid leukemia. Australian leukaemia study group. Blood 84 (4), 1220–1225. 10.1182/blood.v84.4.1220.bloodjournal8441220 8049437

[B7] Chen Z. Yu M. Yan J. Guo L. Zhang B. Liu S. (2021). PNOC expressed by B cells in cholangiocarcinoma was survival related and LAIR2 could be a T cell exhaustion biomarker in tumor microenvironment: characterization of immune microenvironment combining single-cell and bulk sequencing technology. Front. Immunol. 12, 647209. 10.3389/fimmu.2021.647209 33841428 PMC8024580

[B8] Chinese Society of Hematology, Chinese Medical Doctor Association (2018). Chinese guidelines for diagnosis and treatment of acute promyelocytic leukemia (2018). Zhonghua Xue Ye Xue Za Zhi = Zhonghua Xueyexue Zazhi 39 (3), 179–183. 10.3760/cma.j.issn.0253-2727.2018.03.002 29562460 PMC7342995

[B9] de Thé H. Chomienne C. Lanotte M. Degos L. Dejean A. (1990). The t(15;17) translocation of acute promyelocytic leukaemia fuses the retinoic acid receptor alpha gene to a novel transcribed locus. Nature 347 (6293), 558–561. 10.1038/347558a0 2170850

[B10] Dhakal P. Lyden E. Rajasurya V. Zeidan A. M. Chaulagain C. Gundabolu K. (2021). Early mortality and overall survival in acute promyelocytic leukemia: do real-world data match results of the clinical trials? Leukemia and Lymphoma 62 (8), 1949–1957. 10.1080/10428194.2021.1894651 33711907 PMC9429085

[B11] Fang H. Wang S. A. Hu S. Konoplev S. N. Mo H. Liu W. (2022). Acute promyelocytic leukemia: immunophenotype and differential diagnosis by flow cytometry. Cytom. Part B, Clin. Cytom. 102 (4), 283–291. 10.1002/cyto.b.22085 35716019

[B12] Gaafar A. Hamza F. N. Yousif R. Shinwari Z. Alotaibi A. G. Iqniebi A. (2025). Distinct phenotypic and molecular characteristics of CD34-and CD34+ hematopoietic stem/progenitor cell subsets in cord blood and bone marrow samples: implications for clinical applications. Diagn. Basel, Switz. 15 (4), 447. 10.3390/diagnostics15040447 40002599 PMC11853955

[B13] Gupta V. Yib Q.-L. Brandwein J. Chun K. Lipton J. H. Messner H. (2004). Clinico-biological features and prognostic significance of PML/RARalpha isoforms in adult patients with acute promyelocytic leukemia treated with all trans retinoic acid (ATRA) and chemotherapy. Leukemia and Lymphoma 45 (3), 469–480. 10.1080/10428190310001617295 15160908

[B14] Herbrich S. Baran N. Cai T. Weng C. Aitken M. J. L. Post S. M. (2021). Overexpression of CD200 is a stem cell-specific mechanism of immune evasion in AML. J. For Immunother. Cancer 9 (7), e002968. 10.1136/jitc-2021-002968 34326171 PMC8323398

[B15] Huang Q. Zhang Y. Zheng M. (2024). Clinical analysis of 82 cases of acute promyelocytic leukemia with PML-RARα short isoform in children and adults. Front. Oncol. 14, 1342671. 10.3389/fonc.2024.1342671 38450185 PMC10914992

[B16] Infante J. Esteves G. Raposo J. de Lacerda J. F. (2023). Predictors of very early death in acute promyelocytic leukemia: a retrospective real-world cohort study. Ann. Hematol. 102 (11), 3031–3037. 10.1007/s00277-023-05422-z 37650885 PMC10567916

[B17] Kandeel E. Z. Madney Y. Eldin D. N. Shafik N. F. (2020). Overexpression of CD200 and CD123 is a major influential factor in the clinical course of pediatric acute myeloid leukemia. Exp. Mol. Pathology 118, 104597. 10.1016/j.yexmp.2020.104597 33358743

[B18] Li K. Zhang C. Deng J. Zeng H. Zhang Y. Lai G. (2024). Causal effects of gut microbiome on HIV infection: a two-sample Mendelian randomization analysis. BMC Infect. Dis. 24 (1), 280. 10.1186/s12879-024-09176-5 38438963 PMC10913272

[B19] Li P. Liu L. Zhou F. (2024). A distinct subgroup of AML resembling the APL immunophenotype is associated with DIC. BMC Cancer 24 (1), 1576. 10.1186/s12885-024-13348-6 39722004 PMC11670352

[B20] Li D. Lin J. Yang H. Zhou L. Li Y. Xu Z. (2025). Causal association of modifiable factors with cardiometabolic multimorbidity: an exposome-wide Mendelian randomization investigation. Cardiovasc. Diabetol. 24 (1), 241. 10.1186/s12933-025-02790-w 40481456 PMC12143095

[B21] Lin P. Hao S. Medeiros L. J. Estey E. H. Pierce S. A. Wang X. (2004). Expression of CD2 in acute promyelocytic leukemia correlates with short form of PML-RARalpha transcripts and poorer prognosis. Am. J. Clin. Pathology 121 (3), 402–407. 10.1309/XC8P-9M8N-KQDT-38LB 15023045

[B22] Liquori A. Ibañez M. Sargas C. Sanz M. Á. Barragán E. Cervera J. (2020). Acute promyelocytic leukemia: a constellation of molecular events around a single PML-RARA fusion gene. Cancers 12 (3), 624. 10.3390/cancers12030624 32182684 PMC7139833

[B23] Minami M. Sakoda T. Kawano G. Kochi Y. Sasaki K. Sugio T. (2025). Distinct leukemogenic mechanism of acute promyelocytic leukemia based on genomic structure of PML::RARα. Leukemia 39 (4), 844–853. 10.1038/s41375-025-02530-9 39979604

[B24] Murray C. K. Estey E. Paietta E. Howard R. S. Edenfield W. J. Pierce S. (1999). CD56 expression in acute promyelocytic leukemia: a possible indicator of poor treatment outcome? J. Clin. Oncol. Official J. Am. Soc. Clin. Oncol. 17 (1), 293–297. 10.1200/JCO.1999.17.1.293 10458245

[B25] Nixon B. G. Kuo F. Ji L. Liu M. Capistrano K. Do M. (2022). Tumor-associated macrophages expressing the transcription factor IRF8 promote T cell exhaustion in cancer. Immunity 55 (11), 2044–2058.e5. 10.1016/j.immuni.2022.10.002 36288724 PMC9649891

[B26] Ono T. Takeshita A. Kishimoto Y. Kiyoi H. Okada M. Yamauchi T. (2011). Clinical features and prognostic impact of CD56 expression in acute promyelocytic leukemia: long term follow up data from the Japan adult leukemia study Group(JALSG) APL97. Blood 118 (21), 3608. 10.1182/blood.V118.21.3608.3608

[B27] Orfao A. Chillón M. C. Bortoluci A. M. López-Berges M. C. García-Sanz R. Gonzalez M. (1999). The flow cytometric pattern of CD34, CD15 and CD13 expression in acute myeloblastic leukemia is highly characteristic of the presence of PML-RARalpha gene rearrangements. Haematologica 84 (5), 405–412. 10329918

[B28] Rastogi N. Baker S. Man S. Uger R. A. Wong M. Coles S. J. (2020). Use of an anti-CD200-blocking antibody improves immune responses to AML *in vitro* and *in vivo* . Br. J. Haematol. 193 (1), 155–159. 10.1111/bjh.17125 32996123 PMC9851282

[B29] Sandén C. Landberg N. Peña-Martínez P. Thorsson H. Daga S. Puente-Moncada N. (2025). Aberrant expression of SLAMF6 constitutes a targetable immune escape mechanism in acute myeloid leukemia. Nat. Cancer 6, 1821–1838. 10.1038/s43018-025-01054-6 41044242 PMC12643940

[B30] Sanz M. A. Fenaux P. Tallman M. S. Estey E. H. Löwenberg B. Naoe T. (2019). Management of acute promyelocytic leukemia: updated recommendations from an expert panel of the european LeukemiaNet. Blood 133 (15), 1630–1643. 10.1182/blood-2019-01-894980 30803991 PMC6509567

[B31] Senapati J. Kadia T. M. Daver N. G. DiNardo C. D. Borthakur G. Ravandi F. (2025). Therapeutic horizon of acute myeloid leukemia: success, optimism, and challenges. Cancer 131 (7), e35806. 10.1002/cncr.35806 40105906

[B32] Siraj F. Tanwar P. Singh A. Rishi B. Ranjan A. Chopra A. (2021). Analysing “tear-drop” prints of acute promyelocytic leukemia (APML): immunophenotypic prognostication of APML by FCM. Am. J. Blood Res. 11 (4), 446–457. 34540354 PMC8446832

[B33] Slack J. L. Willman C. L. Andersen J. W. Li Y. P. Viswanatha D. S. Bloomfield C. D. (2000). Molecular analysis and clinical outcome of adult APL patients with the type V PML-RARalpha isoform: results from intergroup protocol 0129. Blood 95 (2), 398–403. 10627441

[B34] Wang H.-Y. Gong S. Li G.-H. Yao Y.-Z. Zheng Y.-S. Lu X.-H. (2022). An effective and chemotherapy-free strategy of all-trans retinoic acid and arsenic trioxide for acute promyelocytic leukemia in all risk groups (APL15 trial). Blood Cancer J. 12 (11), 158. 10.1038/s41408-022-00753-y 36404343 PMC9676182

[B35] Xu F. Yin C.-X. Wang C.-L. Jiang X.-J. Jiang L. Wang Z.-X. (2014). Immunophenotypes and immune markers associated with acute promyelocytic leukemia prognosis. Dis. Markers 2014, 421906. 10.1155/2014/421906 25045197 PMC4089198

[B36] Yun J. (2025). Reclassification of acute myeloid leukemia according to the 2022 world health organization classification and the international consensus classification using open-source data. Ann. Laboratory Med. 45 (2), 170–177. 10.3343/alm.2024.0194 39676421 PMC11788709

[B37] Zhang C. Deng J. Li K. Lai G. Liu H. Zhang Y. (2024a). Causal association of monocytes with chronic kidney disease and the mediation role of frailty: a study integrating large-scale two-sample Mendelian randomization and single-cell analysis. Archives Gerontology Geriatrics 123, 105435. 10.1016/j.archger.2024.105435 38583266

[B38] Zhang C. Shi D. Lai G. Li K. Zhang Y. Li W. (2024b). A transcriptome-wide association study integrating multi-omics bioinformatics and Mendelian randomization reveals the prognostic value of ADAMDEC1 in colon cancer. Archives Toxicol. 99 (2), 645–665. 10.1007/s00204-024-03910-3 39680087

