# Supplementary material for: Association between leukemic immunophenotype and overall survival in patients with acute promyelocytic leukemia: a retrospective cohort study
Source: Front Cell Dev Biol. 2026 Jan 14;14:1747649. doi: 10.3389/fcell.2026.1747649 (PMC12846937; doi:10.3389/fcell.2026.1747649)
Supplement: Supplementary file 1 [file DataSheet1.pdf]

Supplementary files

Table S1. The expression of 20 types of immune markers in non-APL-AML group.

Antigen	Positive rate	Non-APL-AML	阳性细胞百分比(%)			
			0%-	25%-50%	50%-75%	75%-
CD7	26.25（100/381）	0.0 (0.0, 21.3), Median (IQR)	77.4	8.7	6.8	7.1
CD117	91.34（348/381）	72.0 ± 30.3, Mean ± SD	12.1	8.4	17.1	62.4
CD33	52.23（199/381）	64.9 ± 33.6, Mean ± SD	16.0	15.5	17.1	51.4
CD10	0.26（1/381）	0.1 ± 1.2, Mean ± SD	100.0	0.0	0.0	0.0
CD34	78.22（298/381）	85.1 (27.5, 95.8), Median (IQR)	24.7	8.1	8.9	58.3
CD19	10.24（39/381）	0.0 (0.0, 0.0), Median (IQR)	92.7	5.0	1.3	1.0
CD13	95.01（362/381）	77.6 ± 26.7, Mean ± SD	6.8	7.6	18.6	66.9
CD11b	21.00（80/381）	0.0 (0.0, 0.0), Median (IQR)	85.0	6.8	4.7	3.4
CD2	2.89（11/381）	0.0 (0.0, 0.0), Median (IQR)	97.6	1.6	0.5	0.3
CD123	89.24（340/381）	68.3 ± 31.7, Mean ± SD	14.2	10.2	18.6	57.0
CD56	41.73（159/381）	0.0 (0.0, 42.9), Median (IQR)	63.5	15.5	7.9	13.1
CD15	37.01（141/381）	0.0 (0.0, 35.1), Median (IQR)	68.8	11.5	10.5	9.2
CD14	7.35（28/381）	0.0 (0.0, 0.0), Median (IQR)	94.5	2.9	0.8	1.8
CD9	11.29（43/381）	0.0 (0.0, 48.0), Median (IQR)	57.7	18.4	11.3	12.6
CD38	93.96（358/381）	81.0 ± 27.9, Mean ± SD	7.1	7.1	10.8	75.1
HLA.DR	87.66（334/381）	74.0 ± 33.4, Mean ± SD	13.6	7.1	10.8	68.5
CD200	63.78（243/381）	47.4 (0.0, 84.1), Median (IQR)	39.4	11.3	18.9	30.4
CD4	18.11（69/381）	0.0 (0.0, 0.0), Median (IQR)	84.5	5.5	5.0	5.0
CD25	18.37（70/381）	0.0 (0.0, 0.0), Median (IQR)	85.0	8.1	6.0	0.8
MPO	79.53（303/381）	58.5 (12.4, 94.8), Median (IQR)	33.4	13.4	9.7	43.4

Table S2. The expression of 20 types of immune markers in PML-RARα (L-type) group.

Antigen	Positive rate	PML-RARα (L-type)	Percentage of positive cells (%)			
			0%-25%	25%-	50%-	75%-100%
CD7	0.00（0/55）	0.0 (0.0, 0.0), Median (IQR)	100.0	0.0	0.0	0.0
CD117	100.00（55/55）	64.2 ± 21.4, Mean ± SD	0.0	32.7	30.9	36.4
CD33	100.00（55/55）	96.8 ± 4.5, Mean ± SD	0.0	0.0	0.0	100.0

CD10	0.00 (0/55)	0.0 ± 0.0, Mean ± SD	100.0	0.0	0.0	0.0
CD34	0.00 (0/55)	0.0 (0.0, 0.0), Median (IQR)	100.0	0.0	0.0	0.0
CD19	0.00 (0/55)	0.0 (0.0, 0.0), Median (IQR)	100.0	0.0	0.0	0.0
CD13	100.00 (55/55)	85.5 ± 15.5, Mean ± SD	0.0	5.5	12.7	81.8
CD11b	0.00 (0/55)	0.0 (0.0, 0.0), Median (IQR)	100.0	0.0	0.0	0.0
CD2	3.63 (2/55)	0.0 (0.0, 0.0), Median (IQR)	96.4	1.8	1.8	0.0
CD123	89.09 (49/55)	64.5 ± 31.1, Mean ± SD	18.2	7.3	23.6	50.9
CD56	12.73 (7/55)	0.0 (0.0, 0.0), Median (IQR)	89.1	1.8	3.6	5.5
CD15	32.72 (18/55)	0.0 (0.0, 27.8), Median (IQR)	70.9	18.2	9.1	1.8
CD14	0.00 (0/55)	0.0 (0.0, 0.0), Median (IQR)	100.0	0.0	0.0	0.0
CD9	100.00 (55/55)	97.5 (95.7, 98.9), Median (IQR)	0.0	0.0	1.8	98.2
CD38	94.55 (52/55)	70.2 ± 25.8, Mean ± SD	5.5	10.9	29.1	54.5
HLA.DR	5.45 (3/55)	2.4 ± 10.6, Mean ± SD	94.5	3.6	1.8	0.0
CD200	3.63 (2/55)	0.0 (0.0, 0.0), Median (IQR)	96.4	3.6	0.0	0.0
CD4	0.00 (0/55)	0.0 (0.0, 0.0), Median (IQR)	100.0	0.0	0.0	0.0
CD25	0.00 (0/55)	0.0 (0.0, 0.0), Median (IQR)	100.0	0.0	0.0	0.0
MPO	100.00 (55/55)	99.5 (99.1, 99.8), Median (IQR)	0.0	0.0	0.0	100.0

Table S3. The expression of 20 types of immune markers in PML-RARα (S-type) group.

Antigen	Positive rate	PML-RARα (S-type)	Percentage of positive cells (%)			
			**0%-25%**	**25%-50%**	**50%-75%**	**75%-100%**
CD7	6.67 (1/15)	0.0 (0.0, 0.0), Median (IQR)	93.3	6.7	0.0	0.0
CD117	100.00 (15/15)	69.9 ± 15.1, Mean ± SD	0.0	6.7	60.0	33.3
CD33	100.00 (15/15)	92.7 ± 16.4, Mean ± SD	0.0	6.7	0.0	93.3
CD10	0 (0/15)	0.0 ± 0.0, Mean ± SD	100.0	0.0	0.0	0.0
CD34	66.67 (10/15)	24.8 ± 20.4, Median (IQR)	46.7	46.7	6.7	0.0
CD19	0 (0/15)	0.0 (0.0, 0.0), Median (IQR)	100.0	0.0	0.0	0.0
CD13	100.00 (15/15)	83.0 ± 19.6, Mean ± SD	0.0	6.7	13.3	80.0
CD11b	6.67 (1/15)	0.0 (0.0, 0.0), Median (IQR)	93.3	6.7	0.0	0.0
CD2	60.00 (9/15)	24.6 (0.0, 35.6), Median (IQR)	53.3	40.0	6.7	0.0
CD123	100.00 (15/15)	80.9 ± 22.6, Mean ± SD	6.7	0.0	26.7	66.7

CD56	20.0（3/15）	0.0 (0.0, 0.0), Median (IQR)	80.0	6.7	13.3	0.0
CD15	13.33（2/15）	0.0 (0.0, 0.0), Median (IQR)	93.3	0.0	0.0	6.7
CD14	0（0/15）	0.0 (0.0, 0.0), Median (IQR)	100.0	0.0	0.0	0.0
CD9	100.00（15/15）	97.5 (95.6, 99.2), Median (IQR)	0.0	0.0	0.0	100.0
CD38	93.33（14/15）	62.6 ± 24.5, Mean ± SD	6.7	20.0	40.0	33.3
HLA.DR	6.67（1/15）	2.2 ± 8.7, Mean ± SD	93.3	3.6	1.8	0.0
CD200	53.33（8/15）	21.7 (0.0, 39.8), Median (IQR)	53.3	26.7	20.0	0.0
CD4	0（0/15）	0.0 (0.0, 0.0), Median (IQR)	100.0	0.0	0.0	0.0
CD25	0（0/15）	0.0 (0.0, 0.0), Median (IQR)	100.0	0.0	0.0	0.0
MPO	100（15/15）	99.4 (99.0, 99.6) Median (IQR)	0.0	0.0	0.0	100.0

Table S4. Subgroup analysis of the effects of CD56 on the survival time of APL patients, adjusted for age, sex, WBC, and DIC.

Subgroup	n.total	n.event_%	crude.HR_95CI	crude.p_value	adj.HR_95CI	adj.p_value	p for interaction
**Age(years)**							
<50	45	8 (17.8)	1.01 (1~1.03)	0.123	1.02 (0.99~1.04)	0.156	0.008
⩾50	27	9 (33.3)	1.07 (1.03~1.11)	0.001	1.07 (1.03~1.12)	<0.001	
**Sex**							
Male	37	7 (18.9)	1.02 (1.01~1.04)	0.009	1.02 (1.01~1.04)	0.01	0.392
Female	35	10 (28.6)	1.01 (0.98~1.04)	0.49	1.01 (0.98~1.04)	0.459	
**WBC (*10^9)**							
<4	44	7 (15.9)	1.02 (1~1.04)	0.107	1.03 (1~1.05)	0.027	0.814
4-10	12	5 (41.7)	1.02 (0.99~1.04)	0.181	1.01 (0.98~1.04)	0.638	
>10	16	5 (31.2)	1.02 (0.98~1.05)	0.354	1.01 (0.98~1.05)	0.463	
**DIC**							
YES	9	8 (88.9)	1 (0.99~1.02)	0.603	1 (0.97~1.02)	0.863	0.399
NO	63	9 (14.3)	1.01 (0.99~1.04)	0.192	1.02 (1~1.05)	0.081	

Table S5. Subgroup analysis of the effects of CD2 on the survival time of APL patients, adjusted for age, sex, WBC, and DIC.

Subgroup	n.total	n.event_%	crude.HR_95CI	crude.p_value	adj.HR_95CI	adj.p_value	p for interaction
**Age(years)**							
<50	45	8 (17.8)	1.06 (1.02~1.1)	0.005	1.05 (1.01~1.09)	0.011	0.633
⩾50	27	9 (33.3)	1.05	0.004	1.05	0.01	

			(1.02~1.09)		(1.01~1.1)		
**Sex**							
Male	37	7 (18.9)	1.06 (1.02~1.1)	0.002	1.06 (1.02~1.1)	0.008	0.399
Female	35	10 (28.6)	1.04 (1.01~1.08)	0.007	1.04 (1.01~1.07)	0.019	
**WBC (*10^9)**							
<4	44	7 (15.9)	1.08 (1.03~1.12)	<0.001	1.05 (1.01~1.1)	0.027	0.17
4-10	12	5 (41.7)	1.02 (0.98~1.06)	0.372	0.99 (0.92~1.07)	0.847	
>10	16	5 (31.2)	1.06 (1~1.13)	0.048	1.08 (1.01~1.17)	0.028	
**DIC**							
YES	9	8 (88.9)	1.03 (0.99~1.06)	0.188	1.02 (0.98~1.07)	0.275	0.819
NO	63	9 (14.3)	1.05 (1.01~1.09)	0.011	1.03 (1~1.07)	0.083	

Table S6. Subgroup analysis of the effects of CD34 on the survival time of APL patients, adjusted for age, sex, WBC, and DIC (For CD34 has invalid values within the WBC range (4-10) *10^9, it was excluded.).

Subgroup	n.total	n.event_%	crude.HR_95 CI	crude. p_value	adj.HR_95CI	adj. p_value	p for interaction
**Age(years)**							
<50	45	8 (17.8)	1.01 (0.97~1.06)	0.531	1.01 (0.97~1.06)	0.533	0.879
⩾50	27	9 (33.3)	1.02 (1~1.05)	0.083	1.02 (0.99~1.05)	0.115	
**Sex**							
Male	37	7 (18.9)	1.06 (1.02~1.1)	0.003	1.06 (1.02~1.1)	0.003	0.015
Female	35	10 (28.6)	1.01 (0.98~1.04)	0.684	1 (0.98~1.03)	0.756	
**WBC (*10^9)**							
<4	44	7 (15.9)	1.03 (1~1.06)	0.042	1.02 (0.98~1.05)	0.316	0.19
>10	16	5 (31.2)	1.03 (0.98~1.08)	0.206	1.04 (0.99~1.09)	0.14	
**DIC**							
YES	9	8 (88.9)	1 (0.96~1.04)	0.964	1.01 (0.96~1.06)	0.698	0.562
NO	63	9 (14.3)	1.02 (0.99~1.05)	0.138	1.01 (0.98~1.04)	0.425	

Table S7. Subgroup analysis of the effects of CD200 on the survival time of APL patients, adjusted for age, sex, WBC, and DIC.

Subgroup	n.total	n.event_%	crude.HR_95 CI	crude. p_value	adj.HR_95CI	adj. p_value	p for interaction
**Age(years)**							
<50	45	8 (17.8)	1.01 (0.96~1.06)	0.708	1.01 (0.96~1.06)	0.835	0.423

⩾50	27	9 (33.3)	1.03 (1~1.05)	0.057	1.02 (1~1.05)	0.093	
**Sex**							
Male	37	7 (18.9)	1.03 (0.99~1.06)	0.109	1.02 (0.99~1.06)	0.187	0.785
Female	35	10 (28.6)	1.03 (0.99~1.06)	0.106	1.02 (0.99~1.05)	0.298	
**WBC (*10^9)**							
<4	44	7 (15.9)	1.05 (1.02~1.09)	0.004	1.04 (1~1.09)	0.054	0.333
4-10	12	5 (41.7)	1 (0.95~1.06)	0.893	0.98 (0.88~1.1)	0.739	
>10	16	5 (31.2)	1.01 (0.97~1.06)	0.627	1.01 (0.96~1.06)	0.728	
**DIC**							
YES	9	8 (88.9)	1.07 (1~1.14)	0.042	1.07 (1~1.15)	0.068	0.184
NO	63	9 (14.3)	1.03 (1~1.06)	0.079	1.02 (0.99~1.05)	0.222	
